# Translational health research: perspectives from health education specialists

**DOI:** 10.1186/2001-1326-1-27

**Published:** 2012-11-08

**Authors:** Holly J Mata, Sharon Davis

**Affiliations:** 1 Hispanic Health Disparities Research Center, The University of Texas at El Paso, 500 University Ave, El Paso, TX, 79968, USA; 2 Department of Public Health Sciences, College of Health Sciences, The University of Texas at El Paso, 500 University Ave, El Paso, TX, 79968, USA

**Keywords:** Translational research, Health promotion, Health education specialists, Health disparities

## Abstract

The phrase “from bench to bedside to curbside” is a common definition of translational research among health disparities researchers. Health Education Specialists can make important contributions to the field of clinical translational medicine, particularly in light of U.S. health care reform and a renewed emphasis on medical home or health care home models.

Health Education Specialists have the training and experience to engage in and facilitate translational research, as well as the opportunity to learn from the translational efforts of other professions and enhance our research, practice, and community partnerships through translational efforts. In this paper, a Translational Health Education Research framework for health education researchers is suggested to foster increased translational efforts within our profession as well as to promote interdisciplinary collaborations to translate a variety of health-related research. A conceptual framework adapted from translational health disparities research that highlights the level and scope of translational research necessary for changes in practice and policy is also provided.

## Translational health research: perspectives from health education specialists

A recent commentary [1] focused on diverse interpretations and definitions of clinical and translational medicine. Health Education Specialists and health disparities researchers have a particular interest in translational health disparities research and community health. Health disparities have been defined as “systematic, plausibly avoidable health differences that adversely affect socially disadvantaged groups” ([2], p. S151). Health equity – the concept of social justice in health – thus involves addressing structural and societal barriers that get in the way of people being able to attain optimal health [2]. The integration of a “social mission” perspective [1] among clinical translational scientists has been prioritized, and it is important for our public health colleagues to consider their own role in the translational research process. The phrase “from bench to bedside to curbside” is a common definition of translational research among health disparities researchers [3]. Health Education Specialists make important contributions to the field of clinical translational medicine, particularly in light of U.S. health care reform and a renewed emphasis on medical home or health care home [4] models.

Health Education Specialists have the training and experience to engage in and facilitate translational research, as well as the opportunity to learn from the translational efforts of other professions and enhance our research, practice, and community partnerships through translational efforts. In this paper, a Translational Health Education Research (THER) framework for health education researchers is suggested to foster increased translational efforts within our profession as well as to promote interdisciplinary collaborations to translate a variety of health-related research. The Responsibility Areas for Entry Level Health Educators [5] provide the foundation for this framework. A conceptual framework adapted from translational health disparities research that highlights the level and scope of translational research necessary for changes in practice and policy is also provided.

Health Education Specialists are credentialed by the National Commission for Health Education Credentialing (NCHEC). Health education is one of the few professions to conduct a role delineation process that led to verified competencies for practice. This process became the foundation for the credentialing of health educators. Just over a decade ago, NCHEC initiated their Competencies Update Project; this project provided a model that outlined entry and advanced-level scopes of practice in terms of professional preparation, credentialing, and professional development [6]. Health Education Specialists from entry level (bachelor’s or master’s degree) to advanced (doctoral degree with substantial health education experience) work in a variety of settings including health care, social services, government, and academic settings. This diversity of experience and the interdisciplinary nature of the profession make health education professionals highly qualified to contribute to translational health research in general, and translational health disparities research in particular.

Translational Research is widely defined and practiced as the “bench-to-bedside” concept outlined by the National Institutes of Health (NIH) [7]. Further elucidation and modeling of medical translational research is highlighted by the National Cancer Institute (NCI) in their translational continuum schematic, which outlines five phases of translational research: basic science discovery, early translation, late translation, dissemination, and adoption [8]. Initially limited to medical applications, translational research has become widespread in scope and practice. Journals devoted to translational research are spurring new analyses and applications of such research within a wide array of disciplines including academia, medicine, public health, and industry [9]. Within our own field, from “bench to bedside to curbside” is a common definition of translational research among health disparities researchers [3].

Translational research is highlighted within the framework of diverse disciplines, including but not limited to social psychology [10], addictive disease [11], diabetes prevention [12], sexual violence [13], and tobacco prevention [14]. Additionally, within the medical field researchers are focusing on honing and refining the conceptualization and contextualization of translational research. Woolf [9] explained the difference between the original “translational blocks (T1 & T2)” as outlined by the Institute of Medicine’s Clinical Research Round table: T1 is commonly understood as the transfer of scientific advances to the development of relevant and applicable clinical research while T2 describes the translation of trial results into practical clinical application.

## Translational research: foundations and new conceptual frameworks

Within the past few years, a third facet of translational research has been identified and integrated into translational medicine discourse. This “T3” builds on T2 and involves the translation of the clinical research into actual dissemination, implementation, and policy [15]. Similarly, health disparities researchers have outlined a parallel four phases of translation, which include discovery, development, delivery, and adoption [3]. This has expanded, reinforced, and made salient the interdisciplinary imperative inherent in translational research; researchers have called for new paradigms of cooperation in which “…the key facilitators of leadership, teamwork, tools, and resources must be established and integrated” ([15], p. 2320). Fleming and colleagues [16] have provided a conceptual framework that highlights the role of translational research in eliminating health disparities, and calls for interdisciplinary collaboration in advancing translational research. Aligning our understanding of translational research with the areas of responsibility for Health Education Specialists can help facilitate translational efforts and can highlight the role of advocacy in research.

**Figure 1:**
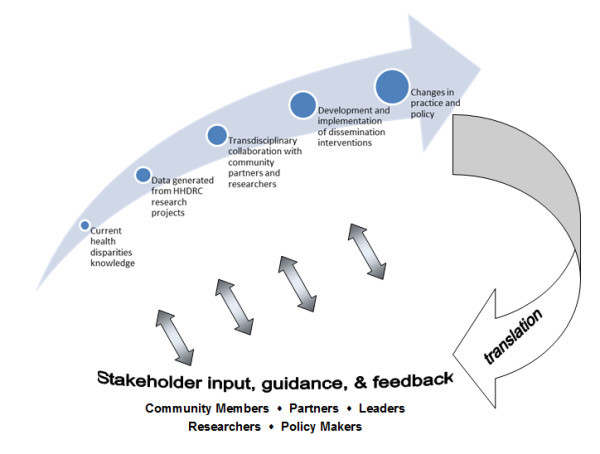
**Conceptual Framework for Translational Research adapted from the translational continuum for health disparities research [**[3]**].**

In past research [17] and in our current work with the Hispanic Health Disparities Research Center (HHDRC) [18], we have suggested that health education researchers have an opportunity and a responsibility to enhance their advocacy efforts through translational research, and that research and advocacy are key components in building health equity. Ongoing work with community partners interested in policy advocacy to build health equity is consistent with our recently developed conceptual framework, which was adapted from an existing translational continuum for health disparities research [3] and tailored to what we and our colleagues envision as inclusive and transdisciplinary translational research (Figure 1). Although the framework was developed to guide our work as health disparities researchers, the process is relevant to anyone engaged in translating research results into changes in practice and policy.

Emerging advances in the environmental health translational research arena have also inspired our work as Health Education Specialists. Building on and parallel to the aforementioned T1, T2, T3 model, Garfin [19] suggested three levels of translational environmental research: TER1 involves basic understanding of “relationships between environmental changes and causes”; TER2 involves “applied environmental research…and development of best practices and decision support tools”; TER3 involves “…impact evaluation…outreach and implementation of best practices…and application of research insights to public policy and institutional change”.

## Proposed Framework for Translational Health Education Research

**Figure 2:**
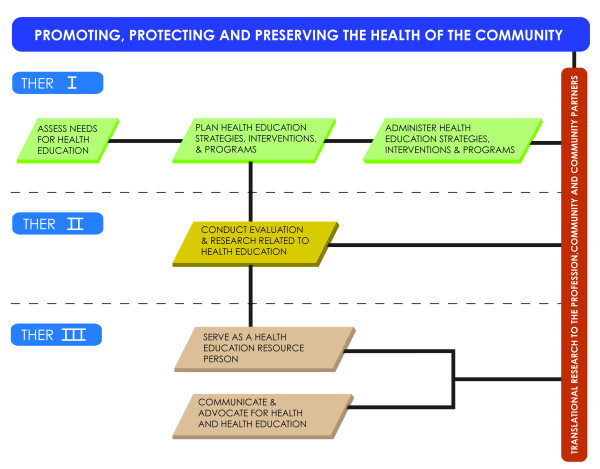
A Framework for Translational Health Education Research.

Following this example, we suggest a Translational Health Education Research (THER) framework for health education researchers to foster increased translational efforts within our profession as well as promote interdisciplinary collaborations to translate a variety of health-related research. The Responsibility Areas for Health Education Specialists [5] provide the foundation (Table 1) for our proposed framework, shown in Figure 2. THER I includes Responsibility Areas I, II, and V. The results of health related assessments must be translated to the profession, and to the community. Proper translation of assessment results can improve the planning and administration of high quality health education programs, strategies, and interventions. Involving the community in the assessment, planning, and administration processes promotes ownership and sustainability of health education programs and is likely to improve intended outcomes [20]. For example, health education programs to reduce smoking by increasing readiness to quit and promoting individual behavior change must also take into account community-level smoking prevalence, attitudes, and social norms.

**Table 1:** **Responsibility Areas for Health Education Specialists**[5]**and alignment with Translational Health Education Research (THER) Framework**

**Areas of Responsibility for Health Education Specialists**	**Examples of Specific Competencies**	**Alignment with THER Framework**
**I. Assess Needs, Assets, & Capacity for health Education**	*Collect health data	THER I
	*Examine relationships between factors that influence health	
**II. Plan Health Education**	*Involve stakeholders	THER I
	*Select strategies/interventions	
**III. Implement Health Education**	*Implement & monitor health education programs	N/A
	*Train individuals involved in health education	
**IV. Conduct Evaluation & Research Related to Health Education**	*Develop evaluation/research plan	THER II
	*Analyze & interpret data	
	*Disseminate research findings	
**V. Administer and Manage Health Education**	*Use communication strategies to obtain support	THER I
	*Promote collaboration among stakeholders	
	*Facilitate partnerships	
**VI. Serve as a Health Education Resource Person**	*Convey health-related information to stakeholders	THER III
	*Establish consultative relationships	
	*Facilitate collaborative efforts to achieve goals	
**VII. Communicate and Advocate for Health and Health Education**	*Analyze the impact of policies on health	THER III
	*Develop a variety of communication strategies	
	*Influence policy to promote health	

THER 2 encompasses Responsibility Area IV. Health education evaluation and research results must be translated within the profession and within communities in such a way as to advance the science of health education and improve policy and practice. Such translation may also enhance the capacity of other practitioners, researchers, and community partners as they refine their research, programming, and/or practice. In historically oppressed and underserved populations, it is often the case that evaluation and research results are not shared with the community in which the research took place [21]. Health Education Specialists have an ethical mandate to address this void and serve the community by translating research results to the community in an effective and culturally relevant manner, as well as in a way that promotes changes in practice and policy.

THER 3 addresses Responsibility Areas VI and VII. The Health Education Specialist must serve as a resource person on interdisciplinary research teams in order to effectively and efficiently disseminate new knowledge and translate research findings. The Health Education Specialist may play a key role in a large academic research center, overseeing community engagement and outreach. Health Education Specialists have the expertise and experience to lead these efforts, which require excellent communication skills to disseminate information in a variety of academic, professional, and community settings. Lastly, the translation of pertinent research findings can be used as an opportunity to advocate for funding for health education programming, additional research funding, and policy change or development based on continuously refined evidence. In other words, *translational research to the profession and to the community* encompasses the last step in the “bench to bedside to curbside” concept. For example, in tobacco control efforts, Health Education Specialists are likely to be involved in reducing tobacco-related health disparities at many levels, including individual behavior change, evaluation of program results, and promotion of comprehensive and coordinated tobacco control policies to reduce smoking and secondhand smoke exposure. Our proposed framework for THER aligns tightly with our conceptual framework for translational health disparities research, and highlights the role of Health Education Specialists in the translational research cycle.

## Enhancing translational capacity

We offer our conceptual framework for translational health disparities research and our framework for translational health education research as guiding principles to improve our translational efforts. More importantly, we hope to engage our colleagues in collaborations that will enhance our collective ability to move research from the “data generated from research projects” phase to the “changes in practice and policy” phase, which will then bring us full circle to translation and new discovery. Health Education Specialists have a responsibility to translate health-related research findings to the profession and to the public to enhance the practice of health education with the ultimate goal of promoting, protecting, and preserving the health of the community. We also have a responsibility to collaborate with our colleagues involved in clinical and biomedical research; these collaborations help make possible the linkages between discovery and delivery, and the cyclical communication that informs translational efforts. In the U.S., the expansion of the primary care workforce propelled by healthcare reform will likely lead to expanded roles and responsibilities for Health Education Specialists in diverse settings. As health education research evolves and generates interdisciplinary collaborative endeavors within the broader scope of public health and medicine, the translational impact will be manifest in better decision-making, better advocacy, better health policy, and better health for all.

## Competing interests

The authors declare that they have no competing interests.

## Author information

HM is a Postdoctoral Research Fellow with the Hispanic Health Disparities Research Center. Her research focuses on health equity policy. She earned a PhD in Interdisciplinary Health Sciences from the University of Texas at El Paso, and is a Certified Health Education Specialist. SD is an Associate Professor with the Department of Public Health Sciences at the University of Texas at El Paso, and is co-director of the Community Engagement and Dissemination Core of the Hispanic Health Disparities Research Center. She earned a PhD in Health Education from the University of New Mexico and is a Master Certified Health Education Specialist.

## Authors’ contributions

Both authors conceptualized the manuscript. HM drafted the manuscript. SD made significant additions and revisions to the manuscript. Both authors conceptualized and designed the frameworks. Both authors read and approved the final manuscript.
